# Cause inhabituelle d'une infection respiratoire récidivante: hypoplasie du poumon gauche

**DOI:** 10.11604/pamj.2013.15.158.2485

**Published:** 2013-08-31

**Authors:** Abdelfettah Zidane, Mohammed Lahkim, AChafik Chafik

**Affiliations:** 1 Service de chirurgie thoracique, Hôpital militaire Avicenne. Marrakech, Maroc; 2 Service de chirurgie viscérale,Hôpital militaire Avicenne. Marrakech, Maroc

**Keywords:** Agénésie pulmonaire, hypoplasie pulmonaire, infection respiratoire, pulmonary agenesis, pulmonary hypoplasia, respiratory infection

## Abstract

L'hypoplasie pulmonaire unilatérale est une malformation congénitale rare qui peut être découverte à l'âge adulte de façon fortuite ou par une infection respiratoire récidivante. Son diagnostic est établi par la tomodensitométrie thoracique avec injection du produit de contraste, et son traitement est essentiellement conservateur. Nous rapportons un cas d'hypoplasie pulmonaire gauche révélée chez un jeune de 20 ans par une infection respiratoire récidivante.

## Introduction

L'hypoplasie est une forme clinique de l'agénésie pulmonaire qui est une malformation congénitale extrêmement rare, définie comme un défaut de développement partiel ou complet du bourgeon pulmonaire primitif. Elle peut être compatible avec la vie dans sa forme unilatérale en l'absence d'autres malformations congénitales associées et/ou de complications respiratoires majeures [1]. Malgré sa rareté, l'hypolplasie pulmonaire unilatérale doit être incluse dans le diagnostic différentiel d'une infection respiratoire récidivante avec opacité radiologique d'un hémithorax. Nous rapportons l'observation d'une hypoplasie du poumon gauche chez un adulte jeune révélée par une infection respiratoire récidivante.

## Patient et observation

**Figure 1:**
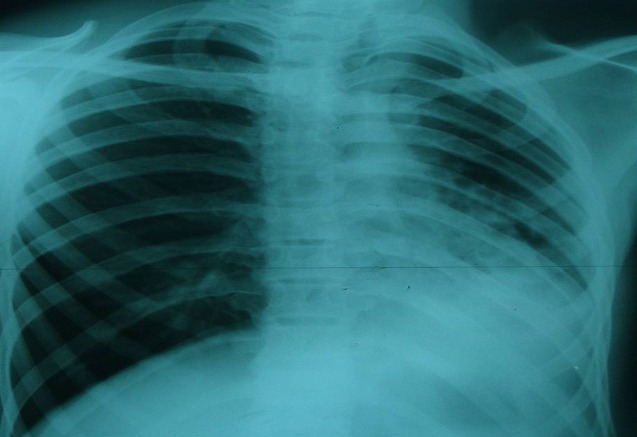
radiographie thoracique de face montrant une opacité basale gauche avec des lésions cavitaires et une déviation médiastinale.

**Figure 2:**
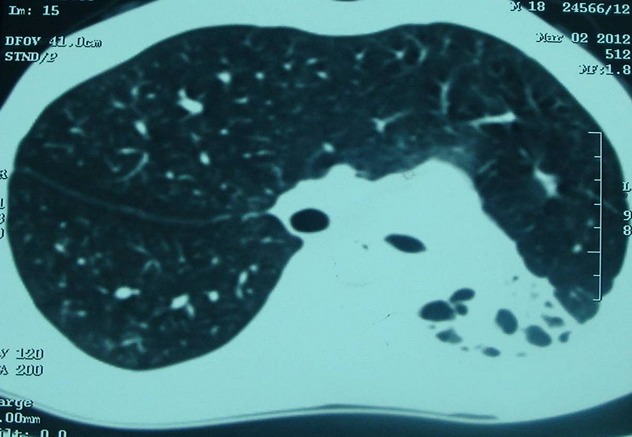
coupe TDM montrant un petit moignon pulmonaire gauche siège de lésions de bronchectasie avec hernie trans-médiastinale importante du poumon droit vers le côté controlatéral

Monsieur O.A âgé de 20 ans, sans antécédents pathologiques particuliers, présentait depuis 4 mois, une bronchorrhée faite de crachats mucopurulents évoluant par poussée et rémission dans un contexte de conservation de l'état général. Par ailleurs, il ne rapportait pas de notion de douleur thoracique ni d'hémoptysie ni de palpitation. A l'examen clinique, il s'agissait d'un patient en bon état général (poids: 53 Kg, taille: 170 cm), eupnéique au repos, présentant des râles bronchiques basithoraciques gauches à l'auscultation. La radiographie thoracique (Figure 1) a objectivé une opacité pulmonaire basale gauche renfermant des images kystiques paracardiaques et une déviation médiastinale vers le côté gauche. La tomodensitométrie thoracique (Figure 2) a montré une importante hernie trans-médiastinale antérieure du poumon droit vers le côté controlatéral, une déviation gauche de la masse cardiaque et des gros vaisseaux du médiastin, une ascension de la coupole diaphragmatique gauche et un moignon pulmonaire gauche rétracté siège de lésions de bronchectasie. La spirométrie a révélé un syndrome mixte, obstructif et restrictif (VEMS: 2,59 L soit 63% de la valeur théorique; CVF: 3 L soit 63%). Un bilan phtysiologique (IDR à la tuberculine, recherche de BK dans les crachats) était négatif. Ainsi, le diagnostic d'hypoplasie pulmonaire gauche a été retenu. Une cure chirurgicale a été proposée au patient qui a refusé. Il était alors mis sous traitement conservateur: antibiothérapie et kinésithérapie respiratoire. L'évolution clinique était favorable avec un recul de 6 mois.

## Discussion

L'agénésie pulmonaire est une malformation congénitale très rare avec une prévalence de 34 par million de nouveau nés [2]. En fonction du stade du développement du bourgeon pulmonaire primitif, elle peut être divisée en trois types [3]:

**Type I:** agénésie pulmonaire bilatérale, incompatible avec la vie.

**Type II:** agénésie pulmonaire unilatérale avec:

1. absence complète du poumon, de la bronche et des vaisseaux sanguins (agénésie)
2. présence d'une bronche rudimentaire mais sans tissu pulmonaire (aplasie)
3. présence d'une bronche rudimentaire, d'une quantité variable de tissu pulmonaire et un support vasculaire (hypoplasie). C'est le cas de notre patient

**Type III:** agénésie lobaire ou autres anomalies.

L'agénésie pulmonaire unilatérale serait plus fréquente du côté gauche, chez l'homme et elle est associée dans plus de 50% des cas à d'autres malformations congénitales cardiovasculaires, gastro-intestinales, génito-urinaires et squelettiques [2, 4]. La moitié des patients décèdent soit à la naissance soit durant les premières années de leur vie du fait de complications broncho-pulmonaires sévères ou de malformations systémiques associées. Son étiologie reste inconnue, mais plusieurs facteurs génétiques, tératogènes et mécaniques ont été impliqués [5].

Sur le plan embryologique, le bourgeon trachéal apparait à partir des cellules ventrales du tube intestinal primitif vers le 28^ème^ jour de gestation, connecté aux deux bourgeons pulmonaires, au même temps que le c'ur bascule vers le côté gauche. L'agénésie pulmonaire unilatérale serait due à une division inégale entre les deux bourgeons pulmonaires, ce qui fait qu'un côté va se développer normalement alors que l'autre va faire complètement défaut (agénésie ou aplasie) ou subir une croissance limitée (hypoplasie).

Chez l'adulte, l'hypoplasie pulmonaire unilatérale peut rester asymptomatique, de découverte fortuite à l'occasion d'une imagerie thoracique réalisée pour autre motif ou un signe en rapport avec une malformation associée, ou se révéler par une infection respiratoire récidivante secondaire à une difficulté de drainage bronchique par distorsion des voies aériennes favorisée par une importante hernie trans-médiastinale du poumon controlatéral et le déplacement du cœur et des structures médiastinales [6].

La radiographie thoracique peut montrer un hémithorax opaque ou une diminution du volume pulmonaire affecté, une hyperinflation du poumon controlatéral et un déplacement médiastinal. Les principaux diagnostics différentiels sont représentés par le poumon détruit, la hernie diaphragmatique congénitale, la malformation adenomatoide kystique et la séquestration pulmonaire. La tomodensitométrie thoracique avec injection du produit de contraste est l'examen de référence pour établir le diagnostic de certitude d'une hypoplasie pulmonaire unilatérale. Elle permet de faire une analyse précise de l'arbre bronchique, du parenchyme pulmonaire et des éléments vasculaires [7]. La bronchoscopie peut être utile pour montrer une bronche rudimentaire.

L'abstention thérapeutique est de mise dans les formes asymptomatiques. Chez les patients présentant une infection respiratoire récidivante, un traitement médical est requis à base de physiothérapie respiratoire active et passive avec drainage de posture et antibiothérapie adaptée dans les poussées infectieuses. Oyamada et al [8] ont rapporté le cas d'un patient de 72 ans vivant avec une agénésie pulmonaire unilatérale bien tolérée. La place de la chirurgie est très limitée, elle est réservée aux échecs du traitement médical ou en cas d'hémoptysie. Elle consiste en la résection d'un moignon bronchique rudimentaire dans les aplasies ou du moignon pulmonaire (pneumonectomie) dans les hypoplasies.

## Conclusion

L'hypoplasie pulmonaire unilatérale est une malformation congénitale rare dont le pronostic dépend de l'intégrité fonctionnelle du poumon restant, d'une part, et des autres anomalies associées d'autre part. Elle peut rester longtemps asymptomatique ou se révéler par des infections respiratoires récidivantes. Son traitement est généralement basé sur des méthodes conservatrices.
